# Supplementary material for: Role of Surgery in Patients with Stage IE Primary Thyroid MALT Lymphoma Staged by a Modified Classification System: The Tokyo Classification
Source: Cancers (Basel). 2023 Feb 24;15(5):1451. doi: 10.3390/cancers15051451 (PMC10000773; doi:10.3390/cancers15051451)
Supplement: Supplementary file 1 [file cancers-15-01451-s001.zip › cancers-2203190-supplementary.pdf]

Suppl. Table S1. Baseline characteristics of the patients with OB-ISRT (*n*=137)

Age, yrs	71 (62.5, 76)
Sex:	
Male	20 (14.6)
Female	117 (85.4)
Coexistence of autoimmune thyroid disease:	
Hashimoto's disease	120 (87.6)
Graves' disease	4 (2.9)
No autoimmune thyroid disease	13 (9.5)
Conventional staging system:	
Stage IE	86 (62.8)
Stage IIE	51 (37.2)
Tokyo classification:	
Stage IE	104 (75.9)
Stage IIE	33 (24.1)
Upstaging after restaging by the Tokyo classification	0
Downstaging after restaging by the Tokyo classification	17

Data are number (%) or median (IQR). OB-ISRT: open biopsy-involved site radiation therapy.

Suppl. Table S2. Comparison of the baseline characteristics and initial treatment characteristics between the OB-ISRT and Surgery groups

	**OB-ISRT** *n*=46	**Surgery** *n*=14	**p**
Age, yrs	68.3 ± 9.1	69.4 ± 10.8	0.701
Sex:			0.077
Male	4 (9)	4 (29)	
Female	42 (91)	10 (71)	
Hashimoto's disease	42 (91)	11 (79)	0.337
Hypothyroidism before treatment	23 (50)	4 (29)	0.223
Lesion location:			0.640
Bilateral lobes	23 (50)	6 (43)	
Unilateral lobe or Isthmus	23 (50)	8 (57)	
Radiation dose, Gy	30.6 (30.6, 33.0)	–	–
Surgical procedure:			–
Total thyroidectomy	–	8 (57)	
Hemithyroidectomy	–	6 (43)	

Data are number (%), mean ± standard deviation, or median (IQR). *$p<0.05$. $^{\dagger}p<0.001$. IQR: interquartile range, OB-ISRT: open biopsy-involved site radiation therapy.

Suppl. Table S3. Details of permanent complications in the patients with OB-ISRT (n=13)

Type:	
Dry mouth	10
Chronic skin problems	3
The timing of symptoms:	
<1 month after the onset of treatment	6
>1 month, <1 yr after the onset of treatment	4
>1 yr after the onset of treatment	3

The data are n-values. All 10 permanent dry mouths started during the first year of ISRT. All three chronic skin problems were reported on the medical record after 1 year following ISRT.

Suppl. Figure S1

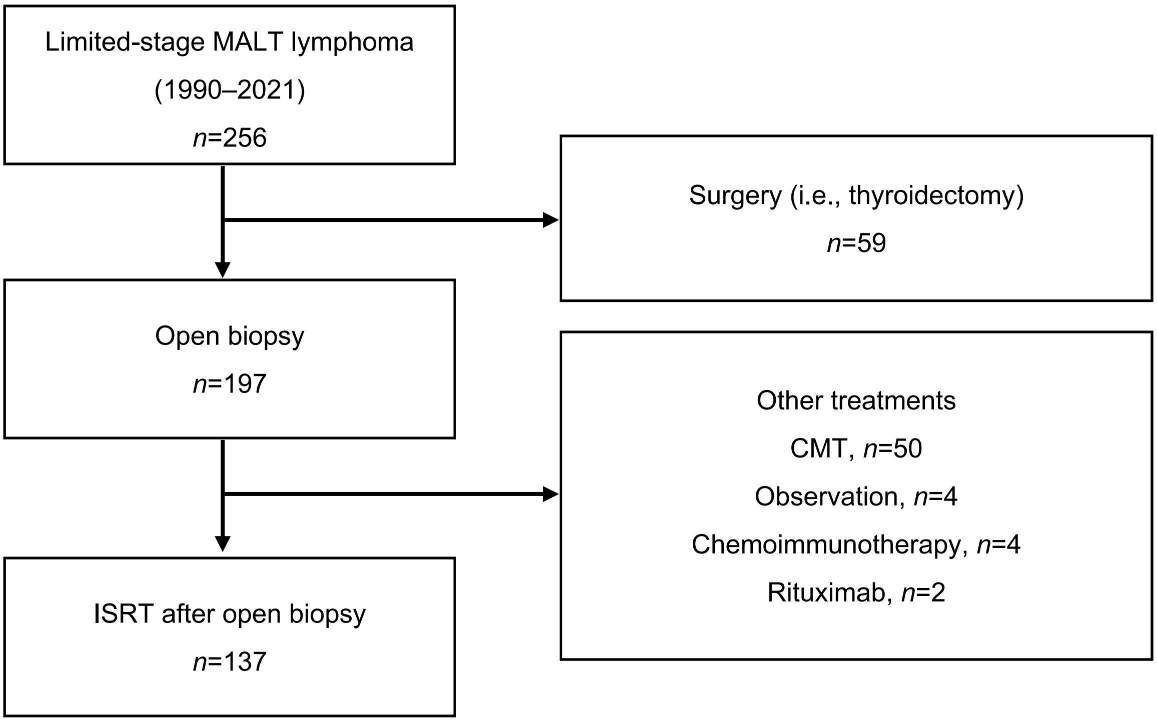

Suppl. Figure S2

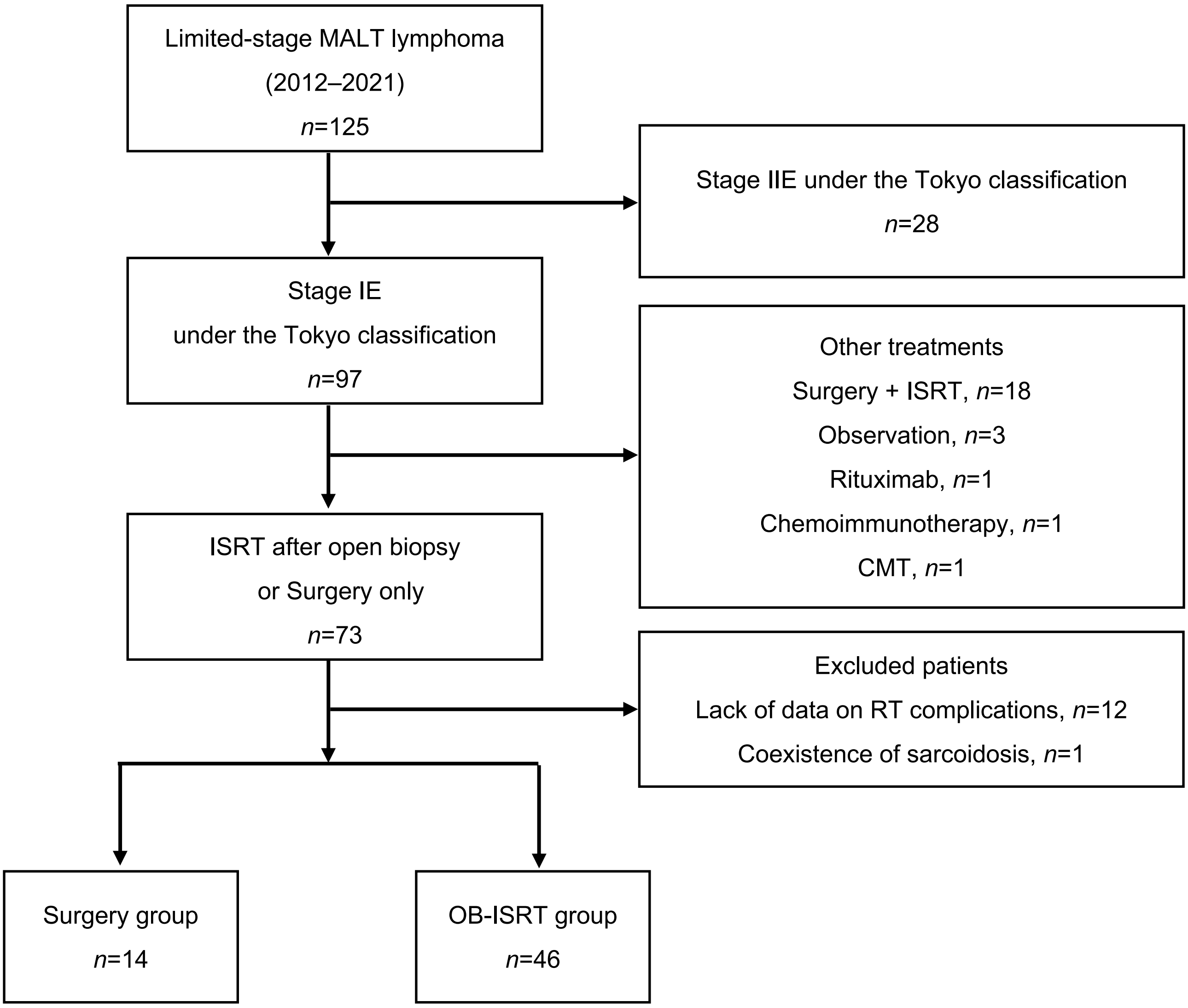

Figure legends

Suppl. Figure S1. Flow chart of patient enrollment in the first study for the assessment of a modified staging system. CMT: combined modality therapy, ISRT: involved site radiation therapy, MALT: mucosa-associated lymphoid tissue.

Suppl. Figure S2. Flow chart of patient enrollment in the second study for the comparison of OB-ISRT and surgery. RT: radiation therapy.
